# N^6^-methyladenosine-modified TRAF1 promotes sunitinib resistance by regulating apoptosis and angiogenesis in a METTL14-dependent manner in renal cell carcinoma

**DOI:** 10.1186/s12943-022-01549-1

**Published:** 2022-05-10

**Authors:** Yuanlei Chen, Zeyi Lu, Chao Qi, Chenhao Yu, Yang Li, Wang Huan, Ruyue Wang, Wenqin Luo, Danyang Shen, Lifeng Ding, Liangliang Ren, Haiyun Xie, Dingwei Xue, Mingchao Wang, Kangxin Ni, Liqun Xia, Jun Qian, Gonghui Li

**Affiliations:** 1 Department of Urology, Sir Run Run Shaw Hospital, Zhejiang University School of Medicine, 3 Qingchun Road, Hangzhou, 310016 China; 2 Department of Clinical Laboratory, Sir Run Run Shaw Hospital, Zhejiang University, Hangzhou, 310016 China; 3 Department of General Surgery, The First Affiliated Hospital of Soochow University, Suzhou, 215006 China; 4 State Key Laboratory of Modern Optical Instrumentations, Centre for Optical and Electromagnetic Research, College of Optical Science and Engineering, International Research Center for Advanced Photonics, Zhejiang University, Hangzhou, 310058 China

**Keywords:** Sunitinib-resistance, TRAF1, METTL14, N6-methyladenosine, RCC

## Abstract

**Background:**

Sunitinib resistance can be classified into primary and secondary resistance. While accumulating research has indicated several underlying factors contributing to sunitinib resistance, the precise mechanisms in renal cell carcinoma are still unclear.

**Methods:**

RNA sequencing and m6A sequencing were used to screen for functional genes involved in sunitinib resistance. In vitro and in vivo experiments were carried out and patient samples and clinical information were obtained for clinical analysis.

**Results:**

We identified a tumor necrosis factor receptor-associated factor, TRAF1, that was significantly increased in sunitinib-resistant cells, resistant cell-derived xenograft (CDX-R) models and clinical patients with sunitinib resistance. Silencing TRAF1 increased sunitinib-induced apoptotic and antiangiogenic effects. Mechanistically, the upregulated level of TRAF1 in sunitinib-resistant cells was derived from increased TRAF1 RNA stability, which was caused by an increased level of N6-methyladenosine (m6A) in a METTL14-dependent manner. Moreover, in vivo adeno-associated virus 9 (AAV9) -mediated transduction of TRAF1 suppressed the sunitinib-induced apoptotic and antiangiogenic effects in the CDX models, whereas knockdown of TRAF1 effectively resensitized the sunitinib-resistant CDXs to sunitinib treatment.

**Conclusions:**

Overexpression of TRAF1 promotes sunitinib resistance by modulating apoptotic and angiogenic pathways in a METTL14-dependent manner. Targeting TRAF1 and its pathways may be a novel pharmaceutical intervention for sunitinib-treated patients.

**Supplementary Information:**

The online version contains supplementary material available at 10.1186/s12943-022-01549-1.

## Background

Renal cell carcinoma is one of the most common cancers worldwide, and its incidence has risen steadily over several decades and is continuing to increase [1, 2]. Surgery is an effective approach and is strongly recommended for patients who present at an early stage [3]. However, approximately 30% of RCC patients have metastatic disease at initial diagnosis [4]. Radiation therapy and chemotherapy are largely ineffective for all RCC subtypes. Therefore, for unresectable RCCs, targeted therapies and immunotherapies are optional strategies [5]. Sunitinib, a multitarget receptor tyrosine kinase (RTK) inhibitor, is a first-line targeted drug for recurrent and unresectable RCC patients and is approved by the NCCN Clinical Practice Guidelines in Oncology [6, 7]. However, approximately 10%–20% of patients with advanced RCC exhibit primary resistance to sunitinib, and most of the remaining patients may develop acquired drug resistance and tumor progression after 6–15 months of therapy [6]. To date, several mechanisms of sunitinib resistance have been indicated, such as lysosomal sequestration of TKIs, angiogenic switching, gene mutations and modifications of gene expression levels and the tumor microenvironment, but the precise mechanisms remain unclear [8]. Therefore, investigating the underlying molecular mechanism of sunitinib resistance in RCC to increase its efficacy is urgently needed.

Tumor necrosis factor receptor (TNFR) associated factor 1 (TRAF1), a signaling adaptor first recognized as a part of the TNFR2 signaling complex, has various roles in human disease [9, 10]. TRAF1, along with TRAF2 and the cellular inhibitors of apoptosis (cIAP1 and cIAP2), is essential for inhibiting TNF-induced apoptosis in NF-κB-deficient cell lines [11]. TRAF1 can augment survival signaling downstream of a subset of TNFR family members through activation of the canonical NF-κB and MAPK pathways [12]. Another study also indicated that TRAF1 was necessary for carcinogenesis in a mouse model of UV-induced skin carcinogenesis [13]. In addition, TRAF1 was found to be upregulated in human non-small-cell lung cancer and its expression level was negatively associated with survival [14, 15]. The results of Patel and Shanmugam indicated that TRAF1 and activation of NF-κB pathway might be involved in paclitaxel sensitivity in breast cancer cells and hormone therapy sensitivity in prostate cancer [16, 17]. Therefore, we hypothesized that TRAF1 might have an effect on sunitinib sensitivity in RCC patients.

N6-methyladenosine (m6A) modification, first described in 1971, has been indicated to influence many steps of mRNA metabolism and has become a common focus in recent years [18, 19]. Methyltransferase-like 3 (METTL3), methyltransferase-like 14 (METTL14), and Wilms tumor 1–associated protein (WTAP) form the core methyltransferase complex [20]. In contrast, fat mass and obesity–associated protein (FTO) and alkB homolog 5 (ALKBH5) function as demethylases to reverse the methylation [21]. Therefore, m6A modification, which is controlled by the functional interplay among these methyltransferases and demethylases, is considered to be dynamic and reversible [22]. Accumulating evidence has indicated that the dysregulated m6A modification in mRNAs or noncoding RNAs plays a critical role in the tumorigenesis and progression in various types of cancers [23–25]. In addition, RNA m6A methylation has been found to be associated with chemotherapeutic resistance, for example, with sorafenib resistance in liver cancer and cisplatin resistance in ovarian cancer and germ cell tumors [26–28]. However, the roles of m6A modification in sunitinib resistance in RCC remain obscure.

In our research, we found that TRAF1 expression was prominently increased in sunitinib-resistant cells, resistant cell derived xenografts and clinical patients with sunitinib resistance. A high expression level of TRAF1 was found to be essential for the maintenance of sunitinib resistance by regulating apoptotic and angiogenic pathways. The increased levels of TRAF1 in sunitinib-resistant RCC cells resulted from its increased TRAF1 mRNA stability which was mediated by elevated m6A modification of specific adenosines in TRAF1. Based on these results, we propose a novel mechanism for sunitinib resistance and suggest that targeting TRAF1 and its pathways may be a novel pharmaceutical intervention for sunitinib-treated patients.

## Materials and methods

### Patient samples

This study was approved by the Ethics Committee of the Sir Run Run Shaw Hospital, Zhejiang university (no.2019021169), and written informed consent was obtained from all patients. All procedures conformed to the principles of the Declaration of Helsinki. 30 tissue samples were obtained from randomly selected sunitinib-treated RCC patients to detect TRAF1 expression and for further immunohistochemistry staining. Low and high TRAF1 expression levels were cut off by median expression values.

### Cell lines and cell culture

The RCC cell line OS-RC-2 (RRID: CVCL_1626) was purchased from China Center of Type Culture Collection (CCTCC, Wuhan, China) and 786–0(RRID: CVCL_1051) was were purchased from National Collection of Authenticated Cell Cutures (NCACC, Shanghai, China). Cells were cultured in RPMI 1640 medium containing 10% fetal bovine serum (Gibco, USA), penicillin (25 units/ml), streptomycin (25 g/ml), 1% L-glutamine. To establish sunitinib-resistant renal carcinoma cell lines, 786-O and OS-RC-2 cells resistant to sunitinib (78R and OSR) were generated by growing sensitive 786-O and OS-RC-2 cells (78S and OSS) cells serially treated with an increasing dose of sunitinib up to 14 μM and 12 μM respectively. After continuous culture in complete medium supplemented with 10 μM sunitinib for > 20 passages, these cells were used as sunitinib-resistant RCC cell lines (78R and OSR) for all subsequent experiments. HUVEC(CVCL_2959) was purchased from the American Type Culture Collection (ATCC, Manassas, VA, USA) and cultured in DMEM medium with 10% fetal bovine serum. All cell lines were mycoplasma negative and identified with authentication reports and were cultured at 37 °C in a humidified incubator with 5% CO2.

### RNA immunoprecipitation (RIP)

The RIP assays were performed by using Magna RIP Kit (Millipore, USA) according to the manufactures’ guidelines. Briefly, 2 × 10^7 RCC cells were harvest and lysed in RIP lysis buffer. After centrifuged at 4 °C, the supernatant was incubated with specific antibodies and negative control IgG at room temperature. Then, the beads-antibody complex was washed and incubated with Proteinase K buffer. The immunoprecipitated RNA was purified and detected by qRT-PCR.

### Intratumoral overexpression or knockdown of TRAF1

The custom-made adeno-associated viral vector carrying full-length cDNA of human TRAF1(AAV9-TRAF1) and its negative control (AAV9-Vector), shRNA for human TRAF1 (AAV9-shTRAF1), and human nonsense control shRNA (AAV9-Control) were purchased from Hanbio Biotechnology Co. Ltd. (Shanghai, China). The adeno-associated virus was injected locally into mice (1 × 10^12 vg/ml, intratumor) to overexpress or knock down TRAF1, respectively. The target sequences of shRNA are listed in Supplementary Table 2.

### Luciferase reporter assay

The wildtype and mutant form of 3’UTR region of TRAF1 was amplified and subcloned into the GV272 backbone (Detailed sequence see Table S3). For the luciferase assay, cells were plated in 24-well plates and co-transfected with dual-luciferase reporter (wt or mut) and METTL14 overexpression plasmid or short interfering RNA using Lipofectamine 2000 (Invitrogen) according to the manufacturer's instruction. Luciferase activity was measured by Dual-Luciferase Assay (YEASEN, Shanghai, China) according to the manufacturer's manual and Renilla luciferase activity was normalized against Firefly luciferase activity.

Additional methods information can be found in Supplementary Materials.

## Results

### Establishment of sunitinib-resistant RCC cell lines and cell derived xenograft models

**Fig. 1:**
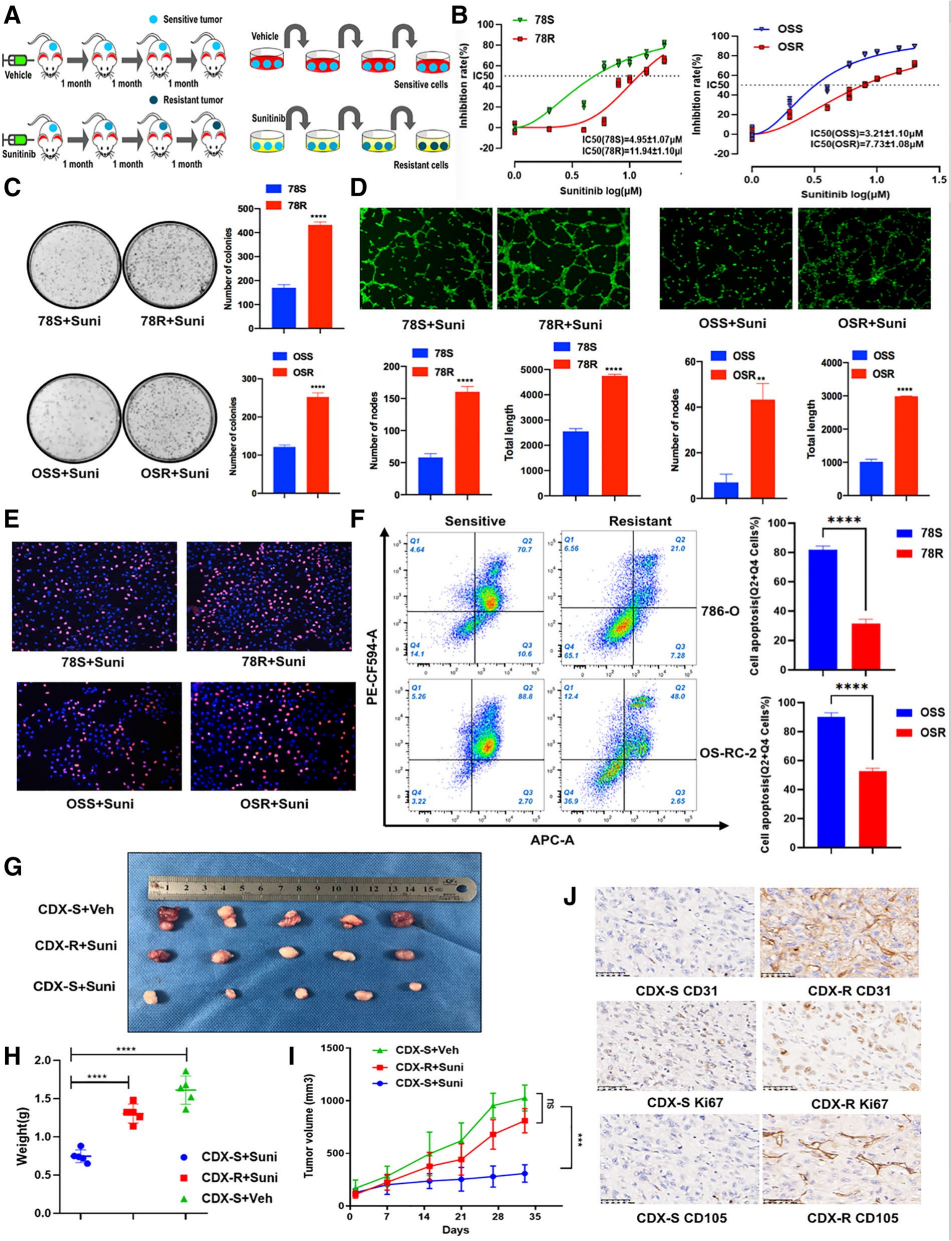
Establishment and verification of sunitinib-resistant models. **A** The graphical representation of sunitinib-resistant models. **B** CCK8 assay of sunitinib-resistant cell lines and control cell lines with sunitinib treatment at indicated concentrations for 48 h. **C** Colony formation assay of sunitinib-resistant cell lines and control cell lines with sunitinib treatment (2 uM) in 6-well dish for 3 weeks (*n* = 3). Representative images (left) and average number of colonies (right) are shown. **D** Tube formation assay of sunitinib-resistant cell lines and control cell lines in 48-well dish. Representative images (up) and total number of nodes and length (below) are shown. **E** EdU assay was applied to compare the cell proliferation ability in sunitinib-resistant cell lines and control cell lines with sunitinib treatment (7 uM for 78S/R, 5 uM for OSS/R) (scale bar, 100 μm). **F** Analysis of apoptosis in 78R, 78S, OSR and OSS cells with sunitinib treatment by flow cytometry (7 uM for 78S/R, 5 uM for OSS/R). **G**-**I** Tumor volume, tumor weight and tumor growth curve of CDX-S and CDX-R under sunitinib or vehicle treatment(40 mg/kg/day) for 30 days. **J** Immunohistochemistry for KI67, CD31 and CD105 comparing CDX-S and control CDX-R. Scale bar, 50 μm. Data are expressed as the mean ± SEM. Statistical analyses used Student’s t-test and Kaplan–Meier survival analysis. *p* < 0.05 was considered statistically significant. * *p* < 0.05, ** *p* < 0.01, *** *p* < 0.001 and **** *p* < 0.0001

To characterize RCC sunitinib resistance in RCC in vitro and in vivo, we established two RCC sunitinib-resistant RCC cell lines (78R and OSR) by chronic exposure to increasing concentrations of sunitinib and established resistant cell derived xenograft (CDX-R) models by oral treatment with sunitinib (Fig. 1A). Compared with the corresponding parental cells (78S and OSS), 78R and OSR cells showed a poor response to sunitinib, as shown by the increased IC50, increased colony formation ability, decreased apoptosis under sunitinib treatment and increased angiogenesis (Fig. 1B-F). However, there was no significant difference in proliferation between the resistant cells and parental cells not treated with sunitinib treatment (Fig. S1A-C). To establish cell derived xenograft models, we implanted 786-O cells into nude mice and treated with mice with sunitinib during three passages in vivo. To verify the resistance of passage 3 xenografts (CDX-R), we implanted CDX-R and CDX-S tumors into nude mice. As shown in Fig. 1G-J, CDX-R tumors showed lower sensitivity to sunitinib treatment than CDX-S tumors, as suggested by the elevated levels of KI67, CD31, and CD105.

### Increased expression level of TRAF1 in sunitinib-resistant RCC

**Fig. 2:**
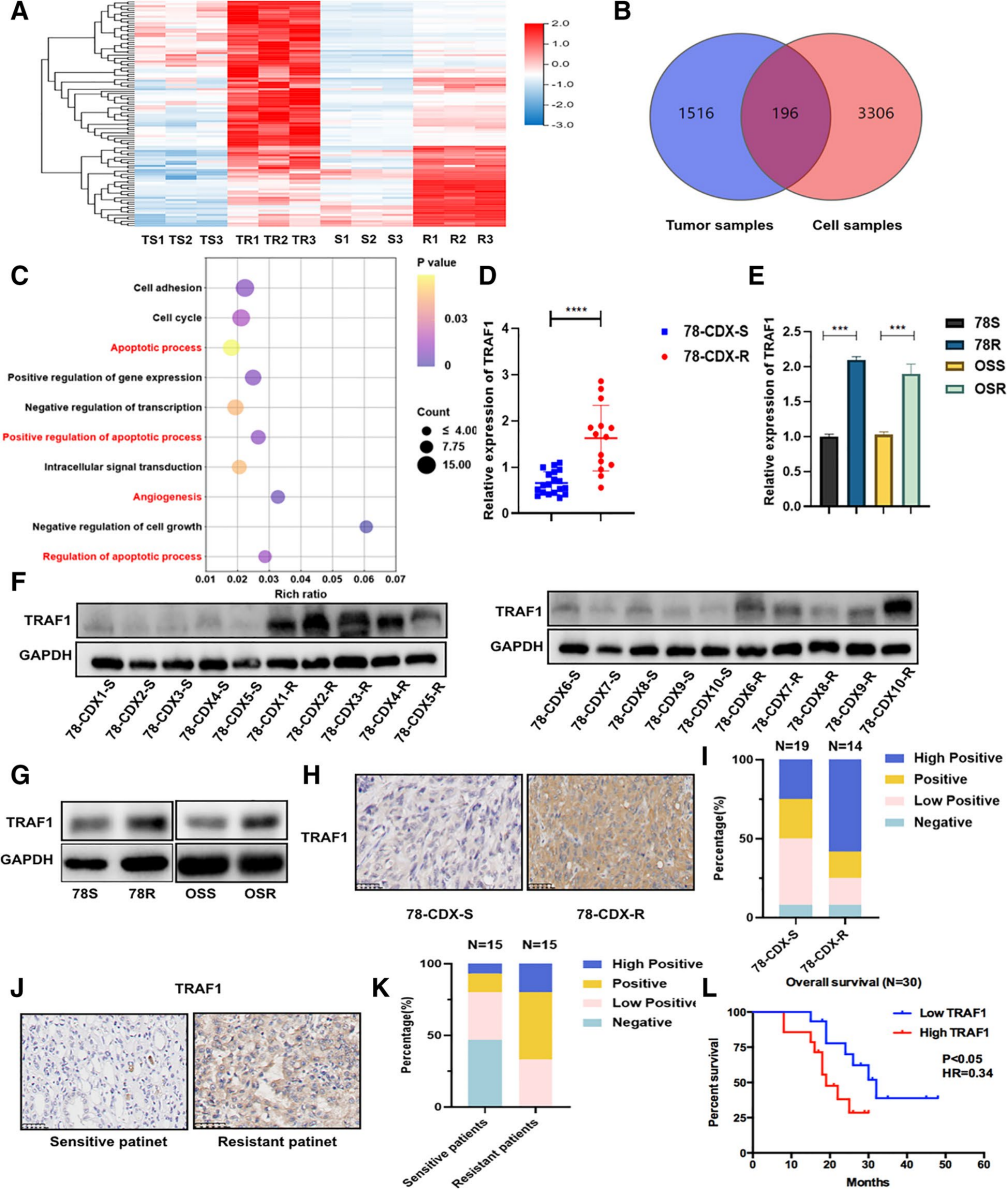
Elevated level of TRAF1 in sunitinib-resistant RCC. **A** Identification of differently expressed genes by RNA sequence in sunitinib resistant cell line and CDX-R compared to the corresponding sensitive groups (TS: sensitive tumor sample; TR: resistant tumor sample; S: sensitive cells; R: resistant cells). **B** The venn diagram was generated from the gene sets enriched for transcripts between tumor samples and cell samples. **C** Gene ontology analysis of differential expressed genes. **D** and **E** The expression of TRAF1 mRNA was determined by RT-qPCR in CDX models and cell lines. **F** and **G** The expression of TRAF1 protein was analyzed by western blotting in CDX samples(*n* = 10) and cell lines. **H** and **I** Immunohistochemistry for TRAF1 in CDX-S and CDX-R. Left panels show images and quantification is shown on the right. **J** and **K** Immunohistochemistry for TRAF1 in clinical patient samples. Left panels show images and quantification is shown on the right. **L** Kaplan–Meier survival analysis for RCC patients treated with sunitinib with low and high TRAF1 expression. The low and high TRAF1 expression was cut off by the median expression. *p* < 0.05 was considered statistically significant. Three different independent experiments with three technical repetitions were performed. Data are expressed as the mean ± SEM. Statistical analyses used Student’s t-test and Kaplan–Meier survival analysis. *p* < 0.05 was considered statistically significant. * *p* < 0.05, ** *p* < 0.01, *** *p* < 0.001 and **** *p* < 0.0001

Three pairs of cells and CDX samples were used for RNA sequencing analysis to investigate the crucial genes involved in sunitinib resistance in RCC (Fig. 2A, Fig. S1D and E). A total of 196 differentially expressed genes were identified and Gene Ontology (GO) enrichment analysis showed that angiogenic and apoptotic pathways may have an effect on sunitinib resistance (Fig. 2B and C). Then, the expression of the top 10 differentially expressed genes involved in angiogenic and apoptotic pathways was verified in both CDX samples and cell lines (Fig. S1F-N). The expression of TRAF1 was significantly upregulated in sunitinib-resistant cells and CDX-R models at both RNA and protein levels (Fig. 2D-G). The same results were also observed by IHC staining of CDX samples (Fig. 2H and I). Consistent with these findings, the frequency of TRAF1-positive cells was higher and the staining intensity was stronger in clinical patients with a poor response to sunitinib (Fig. 2J, K and Supplementary table 1). Importantly, patients with higher expression of TRAF1 showed worse overall survival (Fig. 2L). Collectively, these data implied that TRAF1 was potentially important for sunitinib resistance.

### TRAF1 is crucial for promoting sunitinib resistance

**Fig. 3:**
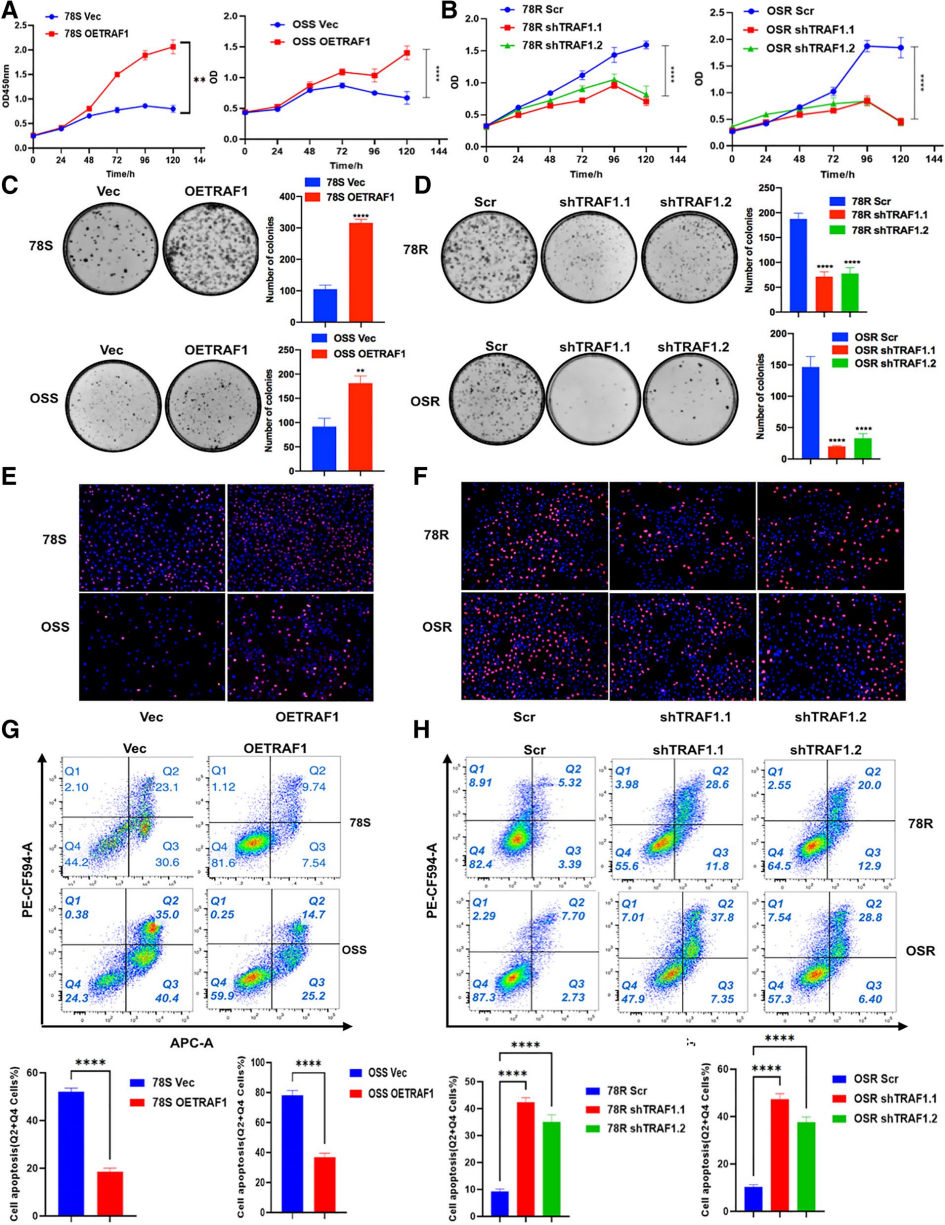
TRAF1 plays a critical role in sunitinib-resistance. **A** CCK8 assay (450 nm) of sunitinib-sensitive cell lines with and without TRAF1 overexpression under sunitinib treatment (5 μM for 78S, 3 μM for OSS) for 5 days. **B** CCK8 assay of sunitinib-resistant cell lines with and without TRAF1 knock-down under sunitinib treatment (5 μM for 78R, 3 μM for OSR) for 5 days. **C** Colony formation assay of sunitinib-sensitive cell lines with and without TRAF1 overexpression under sunitinib treatment (2 μM). **D** Colony formation assay of sunitinib-resistant cell lines with and without TRAF1 knock-down under sunitinib treatment (2 μM). Representative images (left) and average number of colonies (right) are shown. **E** EdU assay of sunitinib-sensitive cell lines with and without TRAF1 overexpression under sunitinib treatment (3 μM). **F** EdU assay of sunitinib-resistant cell lines with and without TRAF1 knock-down under sunitinib treatment (5 μM). **G** Flow cytometry of sunitinib-sensitive cell lines with and without TRAF1 overexpression under sunitinib treatment (3 μM). **H** Flow cytometry of sunitinib-resistant cell lines with and without TRAF1 knock-down under sunitinib treatment (5 μM). Stable expression cell lines were used in the above experiments. * *p* < 0.05, ** *p* < 0.01, *** *p* < 0.001 and **** *p* < 0.0001

To explore the function of TRAF1 in sunitinib resistance, we overexpressed and knocked down the level of TRAF1 in sensitive and resistant cell lines respectively (Fig. S1O-R). Importantly, sunitinib resistance was significantly compromised by TRAF1 knockdown in the RCC sunitinib-resistant cell lines, while increased resistance was found in TRAF1-overexpressing cells (Fig. 3A and B). Analogous results were observed in the colony formation (Fig. 3C and D) and EdU assays (Fig. 3E and F), indicating a critical role of TRAF1 in sunitinib resistance in RCC. In addition, flow cytometric analysis further revealed increased apoptosis in sunitinib-resistant cells upon TRAF1 knockdown (Fig. 3G) and decreased apoptosis in sunitinib-sensitive cells upon TRAF1 overexpression (Fig. 3H).

**Fig. 4:**
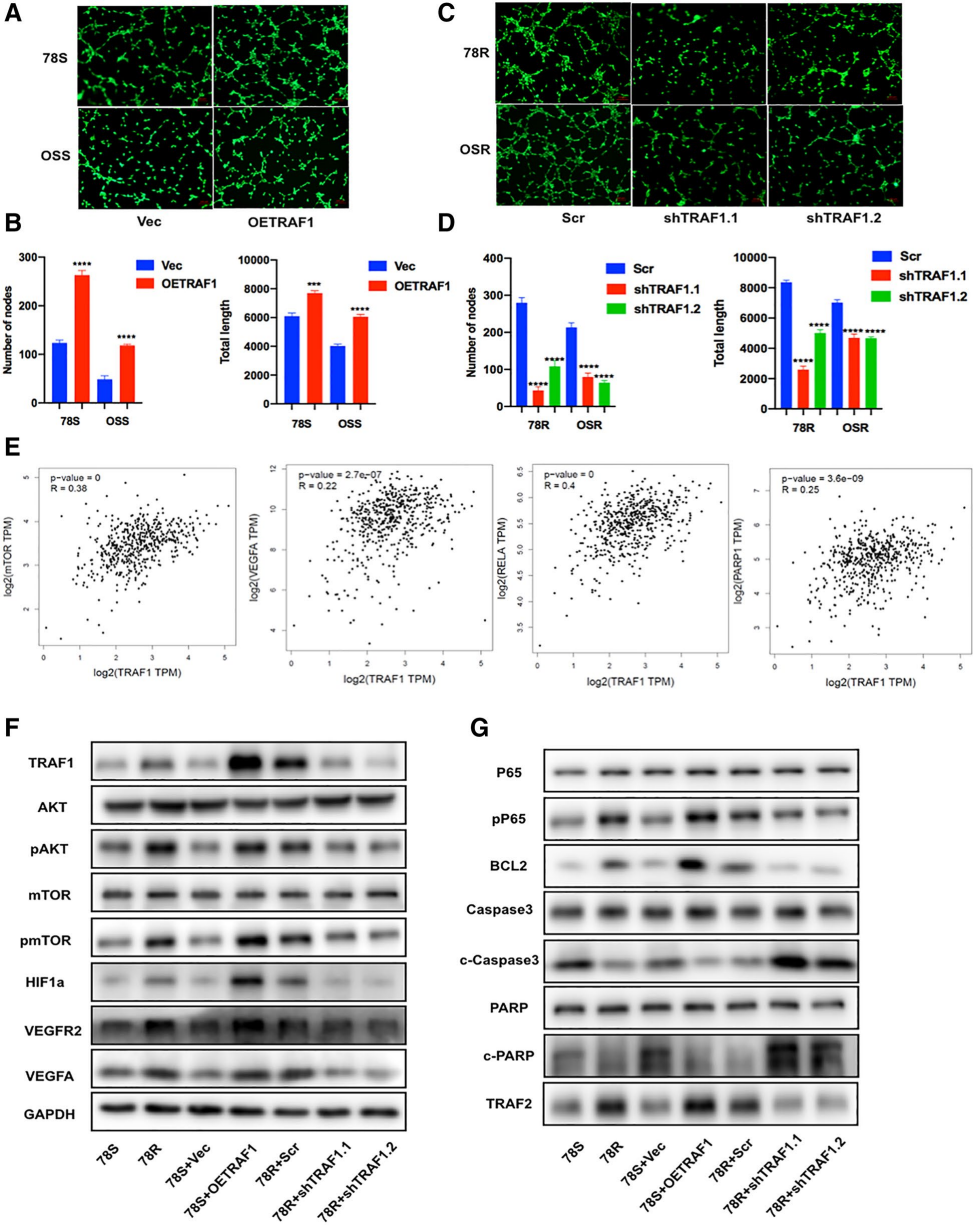
TRAF1 plays a critical role in sunitinib-resistance. **A** and **B** Tube formation assay of sunitinib-sensitive cell lines with and without TRAF1 overexpression under sunitinib treatment. **C** and **D** Tube formation assay of sunitinib-resistant cell lines with and without TRAF1 knock-down under sunitinib treatment. Representative images (up) and total number of nodes and length (below) are shown. **E** Correlation analysis of relative RNA expression of TRAF1 with mTOR, VEGFA, p53 and PARP. **F** Proteins involved in angiogenesis signaling were mediated by TRAF1. **G** Proteins involved in apoptotic signal pathways were mediated by TRAF1

Moreover, the results from the tube formation assay indicated that TRAF1 overexpression markedly enhanced angiogenesis (Fig. 4A and B) and that a reduction in TRAF1 expression suppressed the angiogenesis (Fig. 4C and D). Furthermore, bioinformatics analysis was performed to identify the potential mediators of TRAF1-driven sunitinib resistance (Fig. S2A). We found positive correlations between the relative RNA expression level of TRAF1 and the expression levels of several downstream genes (mTOR, VEGFA, RELA(p65) and PARP) in the TCGA database, indicating the potential association between TRAF1 and these genes (Fig. 4E). Then WB analysis was used to further investigate the specific downstream proteins involved in sunitinib resistance. As shown in Fig. 4F, G and S2D, overexpression of TRAF1 significantly activated the AKT/mTOR/HIF1a/VEGFA pathway. In addition, both the p65 nuclear translocation and the expression of p-p65 were increased with overexpression of TRAF1, indicting the activation of NF-κB pathways and the inhibition of downstream apoptotic pathway. Furthermore, TRAF1 knockdown in resistant cells led to potent inhibition of angiogenic signaling pathways and activation of apoptotic signaling pathways (Fig. 4F, G and Fig. S2D). Similar results were also observed in the OSS and OSR cell lines (Fig. S2B, C and D).

The above results demonstrated that TRAF1 is crucial for maintaining sunitinib resistance and that silencing TRAF1 increases the efficacy of sunitinib by suppressing angiogenesis and inducing tumor cell apoptosis.

### TRAF1 is modulated by m^6^A RNA methylation

**Fig. 5:**
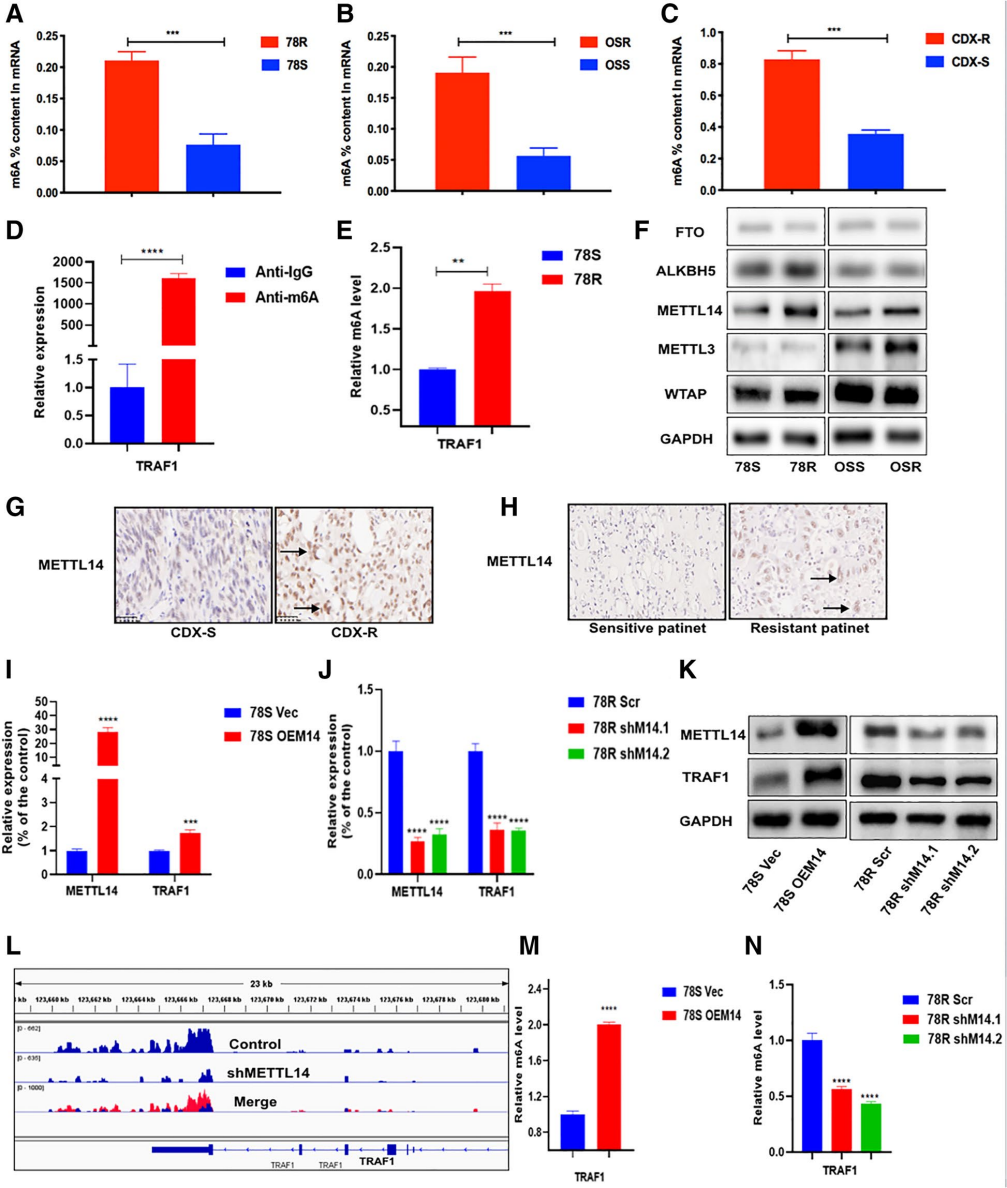
TRAF1 is modulated by m6A RNA methylation. **A** The m6A contents of mRNAs in 78S and 78R cells. **B** The m6A contents of mRNAs in OSS and OSR cells. **C** The m6A contents of mRNAs in CDX-S and CDX-R tissues. **D** and **E** MeRIP assays for m6A-modified TRAF1 in 78S and 78R cells. **F** Protein level of m6A modification–associated genes in sunitinib-sensitive/resistant cells. **G** The protein level of METTL14 in CDX-S and CDX-R. **H** The protein level of METTL14 in clinical patient samples. **I** and **J** The positive correlation of METTL14 and TRAF1 in RNA level. **K** The positive correlation of METTL14 and TRAF1 in protein level. **L** Identification of m6A abundances on TRAF1 transcripts in METTL14 knockdown cell line and control via m6A-sequence. M and N The m6A levels of TRAF1 with modulation of METTL14

In order to investigate whether there is difference in the transcription regulation of TRAF1 between sensitive and resistant cell lines, we performed chromatin immunoprecipitation (CHIP) assay and dual luciferase reporter assay. The results showed no significant difference in transcriptional regulation between sensitive and resistant cell lines (Fig. S3A-C). Previous studies have revealed that m^6^A is the most abundant base modification in RNA and can could modulate the expression of genes in various of cancers [20]. We hypothesized that the upregulated expression of TRAF1 might be modulated by m^6^A modification. Using a colorimetric assay for m6A quantification, we found that m6A levels were significantly upregulated in sunitinib-resistant cell lines and CDX-R samples compared to parental cell lines or CDX-S samples, respectively (Fig. 5A-C). Furthermore, the m6A-specific immunoprecipitation assays displayed up-regulated m6A levels in TRAF1 mRNA in sunitinib-resistant cells compared with wild-type cells (Fig. 5D and E). Recently, numerous catalytic proteins have been identified to participate in dynamic m6A modification. To confirm our hypothesis, we measured the level of m6A catalytic proteins in RCC cell lines. Interestingly, METTL14, a subunit of the N6-adenosine-methyltransferase complex, was significantly upregulated in sunitinib-resistant cell lines (Fig. 5F). However, no significant differences were observed in the expression of FTO, ALKBH5, METTL13 and WTAP. To further characterize the expression of METTL14, we then examined its expression in CDX models and clinical patient samples. As expected, we observed a significant increase in METTL14 expression in sunitinib-resistant tissues (Fig. 5G and H). Moreover, we observed positive correlations between METTL14 and TRAF1 expression at both RNA and protein levels (Fig. 5I-K). Our previous m6A sequencing data (unpublished observation) showed that the m6A levels in TRAF1 mRNA transcripts were decreased in the METTL14-knockdown cell line along with the expression of TRAF1 (Fig. 5J). Consistent with this result, m6A-specific immunoprecipitation assays confirmed that the m6A levels in TRAF1 were reduced or increased when METTL14 was silenced or overexpressed, respectively (Fig. 5M and N). Above all, these results suggested that METTL14 mediates m6A methylation of TRAF1 mRNA and positively modulates TRAF1 expression in sunitinib resistant RCC cells.

### METTL14 regulates the mRNA stability of TRAF1 in an m6A-IGF2BP2-dependent manner

**Fig. 6:**
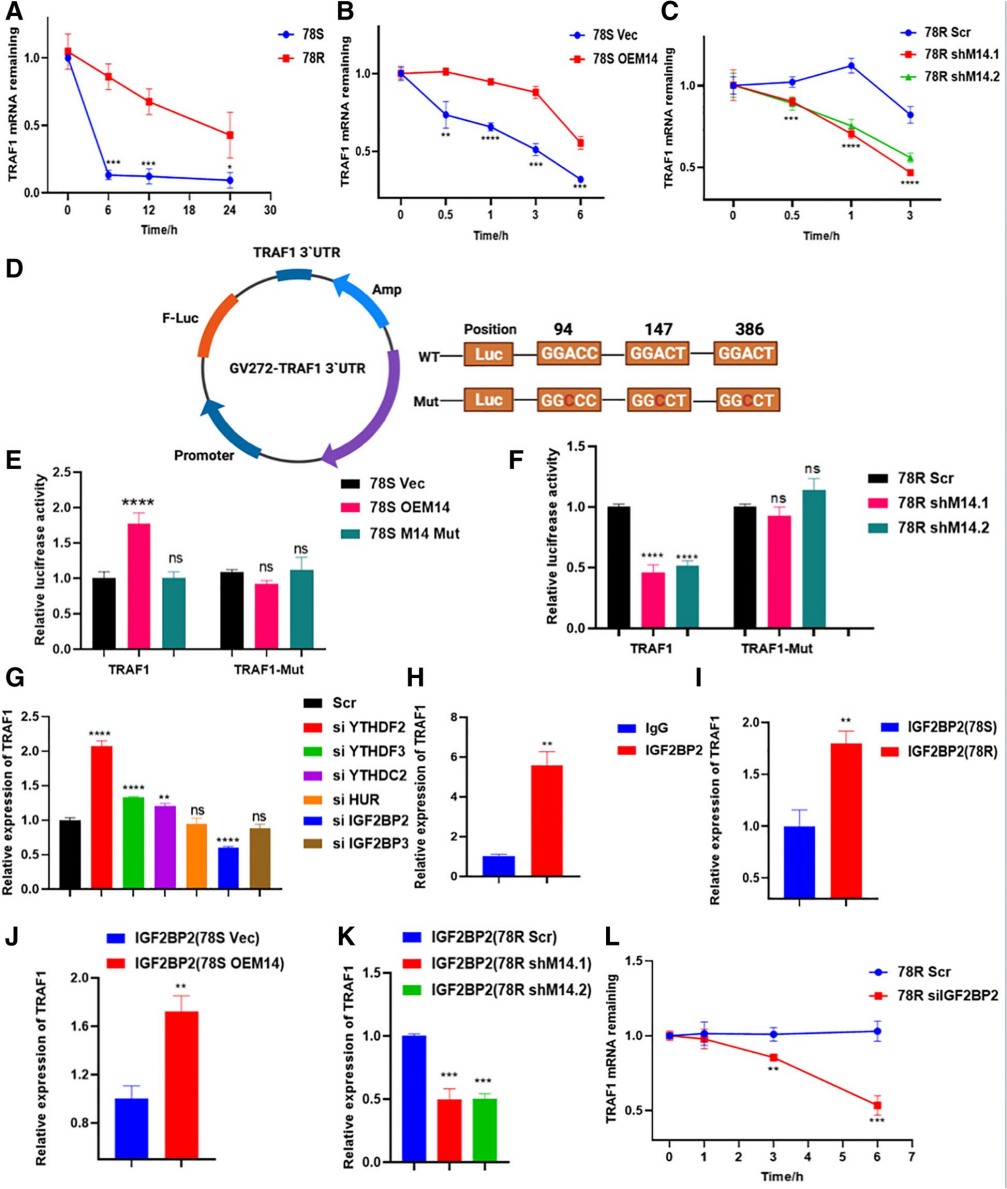
METTL14 regulates mRNA stability of TRAF1. **A** The mRNA half-life (t1/2) of TRAF1 transcripts in 78S and 78R cell lines. **B** The mRNA half-life (t1/2) of TRAF1 transcripts in 78S cells with (OEM14) or without (Vec) METTL14 overexpression. **C** The mRNA half-life (t1/2) of TRAF1 transcripts in 78R cells with (shM14.1 or shM14.2) or without (Scr) METTL14 depletion. **D** TRAF1 3′-UTR plasmid contain wild-type or mutant seed sequences. **E** Relative luciferase activity of TRAF1 3′-UTR constructs containing wild-type or mutant seed sequences after co-transfection with vector (Vec), METTL14 overexpression (OEM14) or mutant METTL14 overexpression (M14 Mut) into 78S cells. **F** Relative luciferase activity of TRAF1 3′-UTR constructs containing wild-type or mutant seed sequences after co-transfection with scramble (Scr) or shMETTL14(shM14) into 78R cells. Firefly luciferase activity was measured and normalized to Renilla luciferase activity. **G** qPCR analysis of the mRNA levels of TRAF1 after knock-down of readers. IGF2BP2 was screened out to positively influence the expression of TRAF1. IGF2BP2 was screened out to positively influence the expression of TRAF1. **H** and **I** RIP assay for the enrichment of TRAF1 in 78S and 78R incubated with IGF2BP2 antibody. TRAF1 is highly enriched in 78R cells compared to 78S cells. **J** and **K** RIP assay for the enrichment of TRAF1 in 78S cells with METTL14 overexpression (**J**) and in 78R cells with METTL14 depletion(K) incubated with IGF2BP2 antibody. **L** The decay rate of mRNA and qPCR analysis of TRAF1 at the indicated times after actinomycin D (5 μg/ml) treatment in 78R cells with IGF2BP2 knockdown

Accumulating evidence has shown that m^6^A peaks on mRNA transcripts can affect mRNA stability. We found that the half-life of TRAF1 transcripts was increased in 78R cells compared with 78S cells (Fig. 6A). To explore whether METTL14 regulates TRAF1 expression by modulating its mRNA stability, cells were treated with the transcription inhibitor actinomycin D (Act D) to measure the half-life of TRAF1 transcripts upon modulation of METTL14 expression. Indeed, overexpression of METTL14 contributed to a noticeable increase in the half-life of TRAF1 transcripts (Fig. 6B), while knockdown of METTL14 led to a significant decrease in the half-life of TRAF1 transcripts (Fig. 6C).

To further support the hypothesis that the regulation of TRAF1 by METTL14 was indeed depends on the methylation of its mRNA transcripts, we constructed luciferase reporter plasmids with the 3’UTR sequence of TRAF1 and the corresponding mutant (Mut-3’UTR) sequence (Fig. 6D). To determine whether the effect of METTL14 is dependent on its ability to recognize m6A targets, we constructed plasmids expressing wild-type METTL14 (METTL14-WT) and mutant METTL14 (METTL14-R298P; R298 is critical for the target recognition of methyltransferase complex [29, 30]). The results of dual luciferase assays showed that WT, but not mutated, METTL14, significantly enhanced the expression of TRAF1 3’UTR reporter (Fig. 6E). In addition, neither wild-type METTL14 nor mutant METTL14 could influenced the luciferase activity of the mut-3`UTR, suggesting the m6A-dependent regulation of RNA stability (Fig. 6E and F).

The modulatory effect of m6A methylation on RNA stability is mediated by two major families of m6A “readers”: the YTH family and the IGF2BP family [31, 32]. To identify the reader participating in the regulation of TRAF1, we designed small interfering RNAs targeting readers reported to enhance RNA stability, among which IGF2BP2 was identified to significantly influence the expression of TRAF1 (Fig. 6G). Importantly, RIP assays showed a direct interaction between the IGF2BP2 and TRAF1 mRNA (Fig. 6H). Additionally, the direct interaction between IGF2BP2 and TRAF1 transcripts was stronger in sunitinib-resistant cell lines (Fig. 6I). Moreover, the interaction was notably affected after modulation of METTL14 expression (Fig. 6J and K). The TRAF1 mRNA stability was impaired in cells with inhibition of IGF2BP2 (Fig. 6L). Together, these findings suggested that METTL14-mediated m6A modification enhances TRAF1 mRNA stability in a IGF2BP2-dependent manner.

### TRAF1 maintained sunitinib resistance in a METTL14-dependent manner

**Fig. 7:**
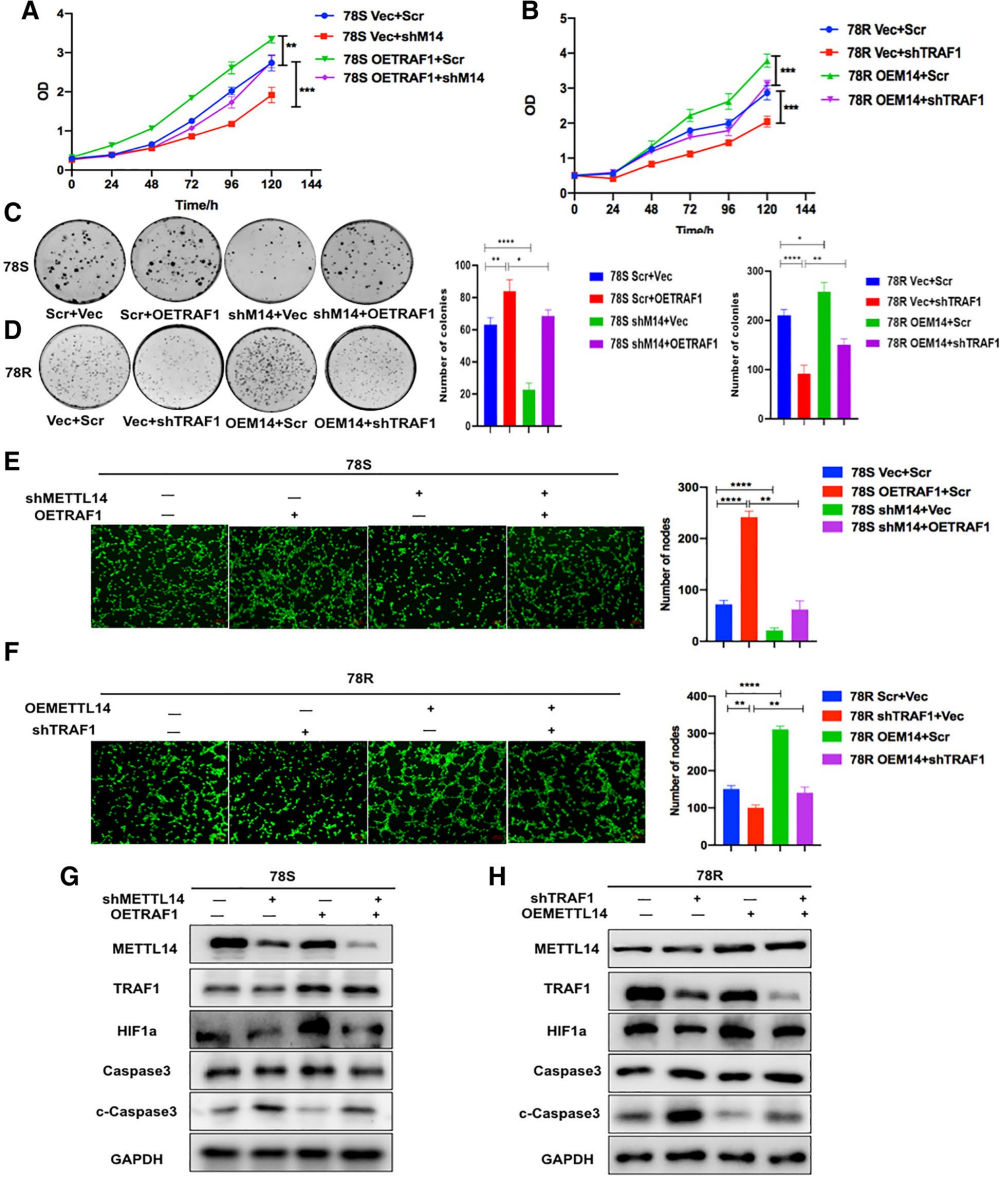
TRAF1 maintained sunitinib resistance in a METTL14-dependent manner. **A** and **B** CCK8 rescue experiments in 78S cells and 78R cells **C** and **D** Colony formation rescue experiments in 78S cells and 78R cells **E** and **F** Tube formation rescue experiments in 78S cells and 78R cells. **G** The protein levels of HIF-1a, Caspase3 and cleaved caspase3 were measured by western blot analysis in 78S cells transfected with lentiviruses carrying TRAF1 and/or sh-METTL14. **H** The protein levels of HIF-1a, Caspase3 and cleaved caspase3 were measured by western blot analysis in 78R cells transfected with lentiviruses carrying METTL14 and/or sh-TRAF1

Based on the above results, we hypothesized that TRAF1 is a functional target of METTL14 in sunitinib resistance. To verify our hypothesis, we performed a series of rescue experiments. The results of CCK-8 (Fig. 7A and B), colony formation (Fig. 7C and D) and tube formation (Fig. 7E and F) assays showed significant inhibition of apoptosis and angiogenesis in METTL14-overexpressing 78R cells, whereas knockdown of TRAF1 diminished the enhancing effect of METTL14 on apoptosis and angiogenesis. In addition, significant enhancement of apoptosis and angiogenesis was observed in METTL14 knockdown cells, and overexpression of TRAF1 restored the antiapoptotic and angiogenic effects of METTL14 knockdown. Moreover, western blot analysis further confirmed that TRAF1 promoted sunitinib resistance by regulating apoptotic and angiogenic pathways in a METTL14-dependent manner (Fig. 7G and H).

### Targeting TRAF1 in vivo suppresses sunitinib resistance in RCC

**Fig. 8:**
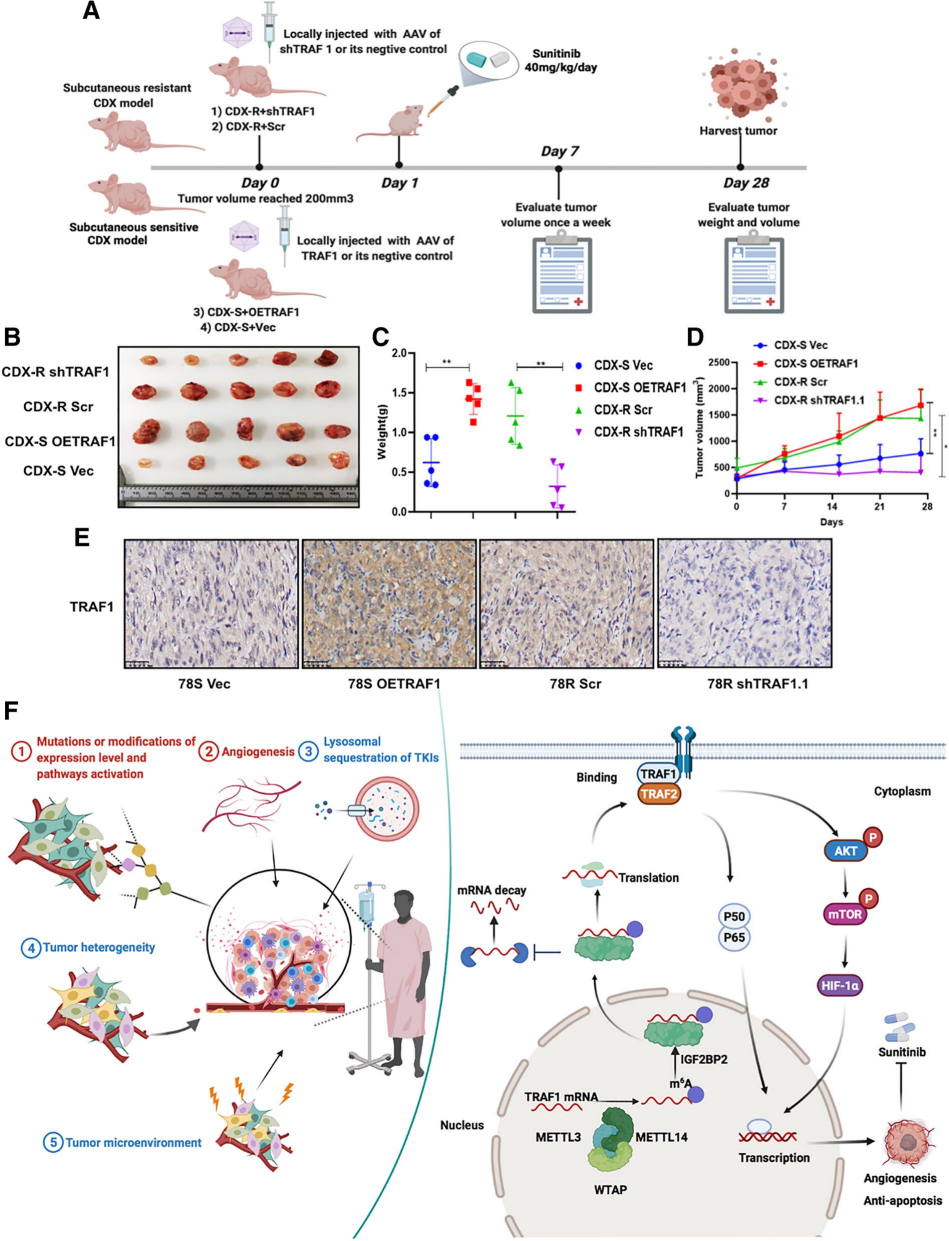
Targeting TRAF1 in vivo retards sunitinib-resistant RCC. **A** Schematic diagram of in vivo experiments. **B**-**D** Tumor volume, tumor weight and tumor growth curve of CDX models with TRAF1 overexpression, TRAF1 knockdown or its relative controls. **E** IHC analysis of the TRAF1 expression. **F** The factors associated with resistance to TKIs (left panel); Proposed model depicting regulation and role of TRAF1 in sunitinib-resistance (right panel)

To further demonstrate our in vitro results and to explore their potential clinical value, we employed in vivo sunitinib resistant models (Fig. 8A). Local injection with AAV of sh-TRAF1 around the subcutaneous implantation site in sunitinib-resistant CDX mice could significantly restore the sensitivity of RCC cells to sunitinib treatment. In contrast, local injection with AAV of OE-TRAF1 promoted sunitinib resistance in CDX-S models (Fig. 8B-D). Consistent with the in vitro findings, IHC staining also showed reduced expression of Ki67, CD31 and CD105 in the sh-TRAF1-treated mice (Fig. S3D), indicating a reduction of antiapoptotic and angiogenic ability in the sh-TRAF1-treated mice (Fig. 8E).

Several factors have traditionally been proposed to be associated with resistance to TKIs, such as gene mutations or modification of gene expression levels and pathway activation, angiogenesis, lysosomal sequestration of TKIs, tumor heterogeneity and the tumor microenvironment. Mechanistically, our results suggested that METTL14-mediated m6A modification enhances TRAF1 mRNA stability to increase the level of TRAF1 in an IGF2BP2-dependent manner. Subsequently, the increased expression of TRAF1 contributes to the activation of downstream antiapoptotic and angiogenic pathways in sunitinib-resistant cells (Fig. 8F). Targeting TRAF1 may be a novel pharmaceutical intervention for sunitinib-treated patients in the near future.

## Discussion

Currently, targeted therapies are the standard treatment options for renal cell carcinoma [33]. In particular, sunitinib is recommended as a first line targeted drug for patients with recurrent and unresectable RCC patients [34, 35]. However, approximately, 10%–20% of advanced RCCs patients are inherently refractory to sunitinib therapy and the majority of the remaining patients will eventually develop drug resistance and disease progression, leding to the failure of sunitinib to efficiently prolong the survival of RCC patients [36, 37]. Therefore, it is essential to explore the underlying mechanism of sunitinib resistance and identify effective targets for its prevention. Currently, numerous mechanisms underlying reduced sensitivity to sunitinib in RCC, including lysosomal sequestration of TKIs, gene mutations and modifications of gene expression levels, the angiogenic switch, constitutive activation of AKT/mTOR signaling, ATP-binding cassette (ABC) efflux transporters, tumor heterogeneity and the tumor microenvironment, have been investigated [38–40]. Accumulating studies have indicated that the activation of compensatory signaling pathways results in the acquisition of sunitinib resistance. Inhibition of PI3K/AKT/mTOR signaling restores sensitivity to sunitinib in ccRCC cells with aberrant AKT activity. However, the overall survival of patients with advanced RCC is still quite unsatisfactory. In our present study, both sunitinib-resistant cell lines and animal models of sunitinib resistance were established to simulate the sunitinib resistance in RCC patients.

Different mechanisms mediate acquired resistance to sunitinib [41]. The identification of reliable biomarkers for the selection of sunitinib-responsive patients and development of appropriate treatment strategies according to the different mechanisms of sunitinib resistance are urgently warranted. Here, we found that the high expression of TRAF1 in RCC before therapy is associated with a poor response to sunitinib. Hence, evaluating the expression of TRAF1 may be helpful to identify patients who could benefit from sunitinib therapy before formulating personalized treatment strategies Recently, AAVs have been increasingly employed to deliver therapeutic genes to in vivo preclinical tumor models. Integrating AAV-mediated gene delivery with traditional treatments (e.g. surgery, chemotherapy and radiotherapy) to formulate novel antitumor strategies is a highly promising approach for future cancer gene therapy [42]. In vivo, local injection with AAV of shTRAF1 suppressed sunitinib resistance in CDX models. In terms of the high expression of TRAF1 in resistant patients, we propose that anti-TRAF1 treatment may increase the clinical curative effect of sunitinib.

TRAF1 is a signaling intermediate for TNFR superfamily members and is known to mainly modulate the NF-κB pathway. Mechanistically, TRAF1 promotes canonical NF-κB activation through cIAP recruitment and possibly through stabilization of TRAF2. In addition, TRAF1 may also result in induction of the alternative NF-κB pathway, also through cIAP recruitment [43]. In our study, we observed that the phosphorylation of P65 and expression of TRAF2 were both changed upon the modulation of TRAF1 expression. Whether TRAF1 activates the canonical NF-κB pathway through stabilization of TRAF2 needs to be further confirmed in future studies.

Many researchers have indicated that m6A modification and the associated regulatory proteins play crucial roles in numerous cancers [23, 44, 45]. As two major components of the m6A MTC, METTL14 and METTL3 have recently been reported to play roles in malignant tumors. METTL14 is required for both the initiation and maintenance of AML and the self-renewal of leukemia stem/initiation cells [46]. In addition, Ma et al. reported that METTL14 plays a tumor suppressor role in hepatocellular carcinoma (HCC), in which METTL14 and m6A levels were found to be decreased compared to those in normal tissue or paratumor control tissues [47]. However, the function of METTL14 in sunitinib resistance is still unknown. In our study, TRAF1 promoted sunitinib resistance by modulating apoptotic and angiogenic pathways in a METTL14-dependent manner. The most well documented m6A readers are the YTH domain-containing proteins [48, 49]. Recently insulin-like growth factor-2 (IGF2) mRNA-binding proteins 1, 2, and 3 (IGF2BP1/2/3) have been identified as a new family of m6A readers that selectively recognize m6A-modified mRNAs with a consensus of GG(m6A)C consensus motif [31, 50]. Interestingly, in our study, we found that IGF2BP1 expression could not be detected in RCC cell lines. However, among several well-known readers that have been reported to potentially promote mRNA stability, IGF2BP2 was identified to positively influence the expression of TRAF1. Moreover, the direct interaction between TRAF1 and IGF2BP2 was notably affected after modulation of METTL14 expression.

Here, we found that the expression of TRAF1 was elevated in sunitinib-resistant models and functionally necessary for the resistance phenotype by regulating apoptotic and angiogenic pathways. The m6A sites in TRAF1 were identified, and the expression of TRAF1 was found to be reduced when its m6A modification was inhibited. Mechanistic analysis suggested that the increased level of TRAF1 was caused by its increased RNA stability, which in turn was caused by an increased level of m6A in a METTL14-dependent manner. Importantly, clinical RCC patients with higher expression of TRAF1 showed poorer responses to sunitinib treatment. However, the number of patient samples in our study was limited, and further demonstration is needed in the future. The specific mechanism of the alterations in the downstream targets of TRAF1 requires further investigation.

## Conclusion

TRAF1 expression was significantly increased in sunitinib-resistant cells, CDX-R models and clinical patients. An elevated level of TRAF1 was imperative for the maintenance of sunitinib resistance via activation of antiapoptotic and angiogenic pathways. The increased level of TRAF1 in sunitinib-resistant RCC resulted from its increased mRNA stability, which was mediated by enhanced N6-methyladenosine (m6A) modification of specific adenosines in TRAF1. Our results provide a new mechanism of sunitinib resistance and indicate that targeting TRAF1 and its pathways may be a novel pharmaceutical intervention for sunitinib-treated patients.

## Supplementary Information

**Additional file 1: Table S2.** Sequences of shRNA&siRNA against specific target in this study. **Fig. S1** A Colony formation assay of sunitinib-resistant cell lines and control cell lines with DMSO in 12-well dish for 3 weeks (n = 3). **Fig. S2 **A TRAF1 pathways in KEGG. B Proteins involed in angiogenesis signaling were mediated by TRAF1 in OS-RC-2 cells. **Fig. S3 **A and B ChIP assays were used to assess the degree of H3K4me3 within the regions 1-3 of the TRAF1 promoter in 78S and 78R cells.

**Additional file 2. **

**Additional file 3: Table1.** TRAF1 intensity of each patients.

## Data Availability

All data used and/or performed in this study are available from the corresponding author upon reasonability.

## Abbreviations

TRAF1: Tumor necrosis factor receptor-associated factor1
METTL14: Methyltransferase-like 14
m6A: N6-methyladenosine
AAV: Adeno-associated virus
CDX: Cell derived xenograft
RCC: Renal cell carcinoma
VEGFR: Vascular endothelial growth factor receptor
TNFR: Tumor necrosis factor receptor
MAPK: Mitogen-activated protein kinase
METTL3: Methyltransferase-like 3
WTAP: Wilms tumor 1–associated protein
FTO: Fat mass and obesity–associated protein
ALKBH5: AlkB homolog 5
