# Supplementary material for: N6-methyladenosine-modified TRAF1 promotes sunitinib resistance by regulating apoptosis and angiogenesis in a METTL14-dependent manner in renal cell carcinoma
Source: Mol Cancer. 2022 May 10;21:111. doi: 10.1186/s12943-022-01549-1 (PMC9087993; doi:10.1186/s12943-022-01549-1)
Supplement: Supplementary file 1 — Additional file 1: Table S2. Sequences of shRNA&siRNA against specific target in this study. Fig. S1 A Colony formation assay of sunitinib-resistant cell lines and control cell lines with DMSO in 12-well dish for 3 weeks (n = 3). Fig. S2 A TRAF1 pathways in KEGG. B Proteins involed in angiogenesis signaling were mediated by TRAF1 in OS-RC-2 cells. Fig. S3 A and B ChIP assays were used to assess the degree of H3K4me3 within the regions 1-3 of the TRAF1 promoter in 78S and 78R cells. [file 12943_2022_1549_MOESM1_ESM.zip › Supplementary Materials 2022.2.docx]

**Supplementary Materials**

**Materials and Reagents**

Sunitinib was purchased from Selleck Chemicals (Houston, TX, USA). Actinomycin D was purchased from MedChemExpress (Monmouth Junction, NJ, USA)

**Western blot analysis**

Cells or tissue samples were lysed in RIPA buffer (Beyotime, Shanghai, China) mixed with loading buffer (Fdbio, Hangzhou, China). And the obtained proteins were separated on 8–12% SDS/PAGE gel and then were transferred into PVDF membranes (Bio-Rad, Hercules, CA, USA). Proteins were incubated with specific primary antibodies and proper HRP-conjugated secondary antibodies after blocking membranes. The blots were visualized by using the ECL system (Bio-Rad, Hercules, CA, USA). The following antibodies were used: anti-TRAF1(# 4715S, Cell Signaling Technology), anti-METTL14(HPA038002, Sigma-Aldrich), anti-CD31(SU03-59,HUABIO), anti-CD105(0902-1,HUABIO), anti-Ki67(ER1802-31,HUABIO),  anti-AKT(ET1609-51,HUABIO), anti-p-AKT(ET1607-73,HUABIO),anti-mTOR(ET1608-5,HUABIO), anti-p-mTOR(#2971S, Cell Signaling Technology), anti-HIF1a(# 14179S, Cell Signaling Technology), anti-VEGFR2(ET1608-33,HUABIO), anti-VEGFA(ET1604-28,HUABIO), anti-P65(ET1603-12,HUABIO), anti-pP65(3033S, Cell Signaling Technology), anti-Bcl-2(# 4223S, Cell Signaling Technology), anti-Caspase3(ER30804,HUABIO), anti-cleaved Caspase3(ET1602-47,HUABIO), anti-PARP(ET1608-56,HUABIO), anti-cleaved PARP(ET1608-10,HUABIO), anti-TRAF2(ET1612-5,HUABIO), anti-GAPDH(ab8245, Abcam), HRP goat anti-rabbit IgG (115-035-003,Jackson ImmunoResearch), HRP goat anti-mouse IgG (111-035-003, Jackson ImmunoResearch), anti-m6A (ab190886, Abcam), anti-IgG (#2729 ,Cell Signaling Technology)

**RNA isolation and Quantitative real‐time PCR (qRT‐PCR)**

Total RNA was extracted by using TRIzol (Cwbiotech, Peking, China) according to the manufacturer’s protocol. cDNA synthesis was performed using a 5X EasyQuick RT MasterMix for qPCR (Cwbiotech, Peking, China). RT-PCR analysis was performed using a 2× SYBR Green qPCR master mix (Cwbiotech, Peking, China). The expression of the target genes was normalized to that of GAPDH. The primer sequences are listed in supplementary Table S1.

**Table1 Sequences of primers in this study**

| METTL14 | forward | TCCCCATAATGATTACTGC |
| --- | --- | --- |
|  | reverse | CTGATGTCAAAGGCTTCTAT |
| GAPDH | Forward | CACATCGCTCAGACACCATG |
|  | Reverse | TTGAGGTCAATGAAGGGGTC |
| TRAF1 | forward | TCCTGTGGAAGATCACCAATGT |
|  | reverse | GCAGGCACAACTTGTAGCC |
| IGF2BP1 | forward | GCGGCCAGTTCTTGGTCAA |
|  | reverse | TTGGGCACCGAATGTTCAATC |
| IGF2BP2 | forward | AGCCTGTCACCATCCATGC |
|  | reverse | CTTCGGCTAGTTTGGTCTCATC |
| IGF2BP3 | forward | TATATCGGAAACCTCAGCGAGA |
|  | reverse | GGACCGAGTGCTCAACTTCT |
| YTHDF2 | forward | CCTTAGGTGGAGCCATGATTG |
|  | reverse | TCTGTGCTACCCAACTTCAGT |
| YTHDF3 | forward | GGTGTATTTAGTCAACCTGGGG |
|  | reverse | AAGAGAACTAGGTGGATAGCCAT |
| YTHDC2 | forward | CAAAACATGCTGTTAGGAGCCT |
|  | reverse | CCACTTGTCTTGCTCATTTCCC |
| HUR | forward | GGGTGACATCGGGAGAACG |
|  | reverse | CTGAACAGGCTTCGTAACTCAT |
| EIF4A3 | forward | GGGGCATCTACGCTTACGG |
|  | reverse | GCGATGACATCTCTCCCTTTGA |

**CCK8, colony formation, EdU and tube formation assays**

CCK8 assay, RCC cells suspended in RPM1640 (10% FBS) were seeded into 96-well dish at a density of 2000 cells per well and were treated with Sunitinib or DMSO after cell attachment. The viability of RCC cells was determined by Cell Counting Kit 8 (YEASEN, Shanghai, China) and measured at OD 450 nm with the BioTek Gen5 system (BioTeck, USA).

Colony formation assay, RCC cells suspended in RPM1640 (10% FBS) were seeded into 6-well dish at a density of 2500 cells per well and were treated with Sunitinib or DMSO after cell attachment. After 2 weeks, colonies were counted using Image J software (NIH Image). The colonies with >50 cells under microscope were counted. Three different independent experiments were performed.

EdU assay, Edu staining proliferation kit was purchased from solarbio (CA1170). The plates were added with EdU solution and were incubated for 2 h and then treated with 4% formaldehyde. After the process, the cells were stained with hochest and apollo solution and performed as the instruction described by Zeiss Axio Observer A1 Inverted Phase Contrast Fluorescence Microscope (ZEISS, Germany).

Tube formation assay, Matrigel was plated at 200 μL/well in 48-well plate at 37℃ for 40 min for hardening. HUVEC cells previously incubated with supernatant from treated cells for 48 hours were harvested and then plated onto the pre-coated Matrigel at a density of 3*10^6^cells per well, and incubated at 37℃ for 4-5 hr. The capillary-like structures were labeled with Calcein AM(2μM) for 30 minutes and photographed using Zeiss Axio Observer A1 Inverted Phase Contrast Fluorescence Microscope (ZEISS, Germany) with 485 nm excitation or 520 nm emission filter.

**PE-Annexin V apoptosis detection**

The apoptosis rate was measured using the Annexin V-APC/PI apoptosis detection kit (AP107-100, liankebio, China). According to the instructions, RCC cells were seeded in 6-well plates and cultured at 37 ℃ for 12 h, and the cells were treated as described under “Cell treatment”. After treatment, RCC cells were collected after digestion by EDTA-free trypsin. The cells were washed twice with pre-cooled PBS, resuspended in 500 μL binding buffer, and incubated with Annexin V-APC and PI for 15 min. The rate of cell apoptosis was measured by flow cytometry (LSR Fortessa, BD, USA).

**RNA sequence and Methylated RNA Immunoprecipitation (MeRIP) sequence**

Total RNA from cells or tissue was isolated using TRIzol (Cwbiotech, Peking, China). For RNA-seq, PolyA-containing mRNA molecules were captured using attached oligo-dT and were subjected to library preparation according to the manufacturer’s instructions. RNA-seq reads were generated by the BGISEQ-500 platform (BGI, Ltd.) with the policy of single-ended 50 bp (SE50). The average RNA-seq depth was 24 million reads, ranging from 23.9–24.1 million reads. Clean reads were mapped to the hg19 genome with TopHat software (v1.4.1, cole-trapnell-lab.github.io/projects/tophat/). The transcription levels (RPKM) were quantified with Cufflink software (v2.0.2, cole-trapnell-lab.github.io/projects/cufflinks/). Fold changes of gene expression were calculated with Cuffdiff software (v2.2.1, cole-trapnell-lab.github.io/cufflinks/cuffdiff/).

For MeRIP-seq, total RNA was isolated using TRIzol. RNA samples were assayed for global m6A levels using an m6A RNA Methylation Quantification Kit (EpiGentek). mRNA was further purified using the Dynabeads mRNA DIRECT kit (Thermo Fisher, MA, USA) and fragmented by sonication. MeRIP-seq and library preparation were performed per the reported protocol (19) with some modifications. Briefly, sonicated mRNA was mixed with m6A antibody (Synaptic Systems, 202003) in IP buffer and incubated with head-to-tail mixing at 4 °C for 2 h. The mixture was supplemented with Protein A magnetic beads (Thermo Fisher, MA, USA) and incubated with head-to-tail mixing at 4 °C for another 2 h. The beads were then washed with IP buffer three times before eluted with m6A elution buffer for two times. The eluates were combined and purified with RNA Clean and Concentrator (Zymo, Orange, CA). The purified mRNA fragments were then used to construct libraries with TruSeq Stranded mRNA Library Prep Kit (Illumina, San Diego, CA). Sequencing was carried out on Illumina HiSeq 2000 with pair-end 150-bp read length. Reads were aligned to human genome version 38 (GRCh38) with Tophat. The longest isoform was retained if a gene has more than one isoform. Differential m6A modified peaks between IP and input samples were identified using exomePeak (p < 0.01).

**Cell transfection**

Short interfering RNA (siRNA) sequences were directly synthesized (RiboBio, Guangzhou, China). The siRNAs were transfected into cells using Lipofectamine RNAiMAX transfection reagent (Invitrogen, Carlsbad, CA, USA) according to manufacturer’s guidance. Ectopic expression plasmids of indicated genes were synthesized by GENECHEM (Shanghai, China) and transfected using Lipofectamine 3000 (Invitrogen, Carlsbad, CA, USA). Lentivirus was synthesized by GENECHEM (Shanghai, China), and infected RCC cells with 5mg/mL polybrene for 3 days according to manufacturer’s instruction. Stable infected cell lines were selected using puromycin (Selleck, Shanghai, China). The shRNA and siRNA sequences are listed in supplementary Table S2.

**Table2 Sequences of shRNA&siRNA against specific target in this study**

| shRNA&siRNA |  | Target sequence |
| --- | --- | --- |
| METTL14 shRNA01 | 5’-3’ | CAAAGATGAGCAGAGAGAAATTGCT |
| METTL14 shRNA02 | 5’-3’ | GCATTGGTGCCGTGTTAAA |
| TRAF1 shRNA01 | 5’-3’ | CTTCTACACTGCCAAGTAT |
| TRAF1 shRNA02 | 5’-3’ | GAACCCATCTGTCGCTCTT |
| IGF2BP1 siRNA | 5’-3’ | AAGCTGAATGGCCACCAGTTG |
| IGF2BP2 siRNA | 5’-3’ | CCGGGAGCAGACCAGGCAA |
| IGF2BP3 siRNA | 5’-3’ | AATCGATGTCCACCAGTAAAGA |
| YTHDF2 siRNA | 5’-3 | AAGGACGTTCCCAATAGCCAA |
| YTHDF3 siRNA | 5’-3 | ATGGATTAAATCAGTATCTAA |
| YTHDC2 siRNA | 5’-3 | GCCTTGGATGTAAATCTCTTT |
| HUR siRNA | 5’-3 | GGTTGCGTTTATCCGGTTT |

**H&E and immunohistochemical (IHC) staining**

Tissues were fixed in 10% (v/v) formaldehyde in PBS, embedded in paraffin, and cut into 5 μm sections and used for H&E staining and IHC staining with specific primary antibodies. To enhance antigen exposure, the slides were treated with 1 × EDTA at 98°C for 10 minutes for antigen retrieval. The slides were incubated with endogenous peroxidase blocking solution, and then were incubated with the primary antibody at 4 ℃ overnight. After rinsing with Tris-buffered saline, the slides were incubated for 45 minutes with biotin-conjugated secondary antibody, washed, and then incubated with enzyme conjugate horseradish peroxidase (HRP)-streptavidin. Freshly prepared DAB (Zymed, South San Francisco, CA) was used as substrate to detect HRP. Finally, slides were counter-stained with hematoxylin and mounted with aqueous mounting media. Positive cells were calculated as the number of immunopositive cells × 100% divided by total number of cells/fields in 10 random fields at 400 × magnification. The slides were reviewed and scored by an experienced pathologist without the knowledge of patient outcome. The staining results were measured semiquantitatively.

**In vivo studies**

4-6 weeks old female athymic BALB/c nude mice (SLAC-Shanghai Laboratory Animal Center, China) were fed in standard pathogen-free conditions. All surgeries were performed under anesthesia. Mice weight and xenograft volumes (Volume = a × b2/2, a represents long axis and b represent short axis) were measured weekly.

To obtain sunitinib-resistant subcutaneous cell derived xenograft (CDX) model, 10^7^ 786-O cells mixed with Matrigel (1:1) were injected into the flanks of 4–6-week-old female BALB/c nude mice. When the volume of xenograft reached 200 mm3, mice were orally treated with vehicle or sunitinib (40 mg/kg/day) for 4 weeks. After one treatment, the most resistant xenografts were isolated and physically dissected into 1 mm3 tissue blocks to sub-transplant into the flank of 4–6-week-old female BALB/c nude mice for the next generation followed with vehicle or sunitinib treatment. CDX model treated with sunitinib from the 3rd generation xenografts were isolated and confirmed to be sunitinib-resistant (termed as CDX-R), and the CDX models treated with vehicle for three generations were termed as CDX-S.

CDX-S and CDX-R xenografts were both disaggregated into 1mm3 tissue blocks and transplanted into 10 mice separately. When the subcutaneous xenografts reached approximately 200 mm3 (four weeks), the mice were randomized into four groups (5 mice in each group): 1) CDX-S treated with AAV9-Vector; 2) CDX-S treated with AAV9 overexpression TRAF1; 3) CDX-R treated with AAV9-Control and 4) CDX-R treated with AAV9-shTRAF1. All the mice were treated with sunitinib (40 mg/kg/mouse; daily, oral gavage) for 4weeks and each tumor was locally injected with AAV9 or its negative control according to the group. Mice were euthanized on the fifth week and xenografts were isolated for further studies.

All animal experiments were performed humanely in compliance with guidelines reviewed by the Animal Ethics Committee of the Biological Resource Centre of the Agency for Science, Technology and Research at the Sir Run-Run Shaw Hospital.

**Statistical Analysis**

Data are expressed as mean ± SEM from at least three independent experiments. Statistical analyses used Student’s t-test, Kaplan-Meier survival analysis and log-rank test with GraphPad Prism 8 (GraphPad Software, Inc., La Jolla, CA). P <0.05 was considered statistically significant. Gene Ontology Analysis was performed online by Database for Annotation, Visualization and Integrated Discovery (DAVID) bioinformatics resources (v6.8)

**Supplementary Fig.1** A Colony formation assay of sunitinib-resistant cell lines and control cell lines with DMSO in 12-well dish for 3 weeks (n = 3). Representative images (left) and average number of colonies (right) are shown. B EdU assay was applied to compare the cell proliferation ability in sunitinib-resistant cell lines and control cell lines with DMSO treatment (scale bar, 100 μm). C Analysis of apoptosis in 78R and 78S cells by flow cytometry. D and E Volcano plot of differentially expressed genes in cell line and tissue samples. F-N The expression of NME4, TAM2, IRF5, NLRP1, CLU, UBD, GPER1, FHIT and WWOX mRNA were determined by RT-qPCR in CDX models. O-R The efficacy of TRAF1 overexpression was measured in 78S and OSS cells. O-P The efficacy of TRAF1 knockdown was measured in 78R and OSR cells.

**Supplementary Fig.2** A TRAF1 pathways in KEGG. B Proteins involed in angiogenesis signaling were mediated by TRAF1 in OS-RC-2 cells. C Proteins involed in apoptotic signal pathways were mediated by TRAF1 inOS-RC-2 cells. D Western blot analysis of the nuclear and cytosol fractions.

**Supplementary Fig.3** A and B ChIP assays were used to assess the degree of H3K4me3 within the regions 1-3 of the TRAF1 promoter in 78S and 78R cells. C Dual luciferase reporter assays were used to assess the luciferase activity of promotor luciferase reporter plasmid in sensitive and resistant cells. D IHC analysis of the CD31, CD105, KI67 protein expression.
