# Supplementary figures and images for: N6-methyladenosine-modified TRAF1 promotes sunitinib resistance by regulating apoptosis and angiogenesis in a METTL14-dependent manner in renal cell carcinoma
Source: Mol Cancer. 2022 May 10;21:111. doi: 10.1186/s12943-022-01549-1 (PMC9087993; doi:10.1186/s12943-022-01549-1)

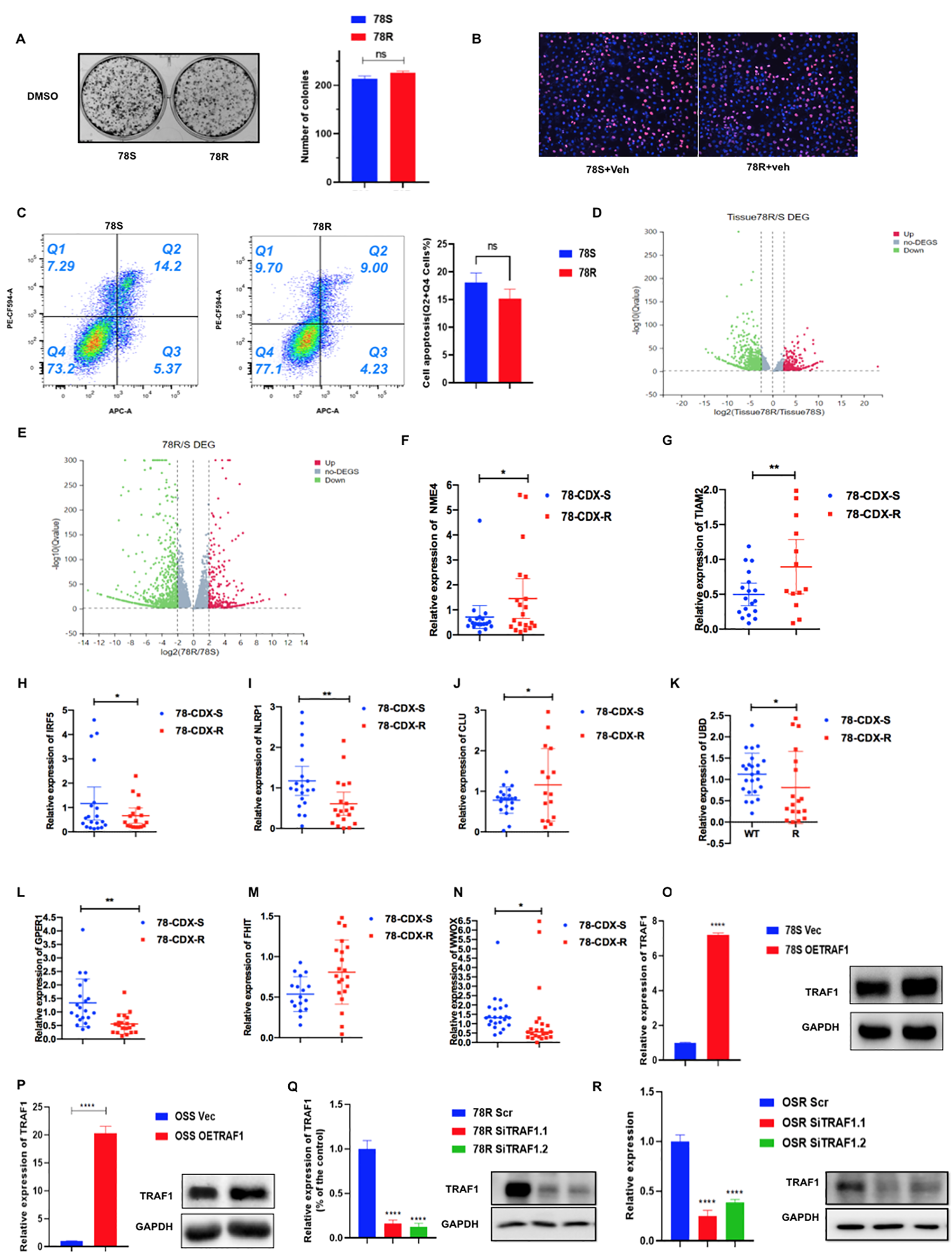

Supplement: Supplementary file 1 — Additional file 1: Table S2. Sequences of shRNA&siRNA against specific target in this study. Fig. S1 A Colony formation assay of sunitinib-resistant cell lines and control cell lines with DMSO in 12-well dish for 3 weeks (n = 3). Fig. S2 A TRAF1 pathways in KEGG. B Proteins involed in angiogenesis signaling were mediated by TRAF1 in OS-RC-2 cells. Fig. S3 A and B ChIP assays were used to assess the degree of H3K4me3 within the regions 1-3 of the TRAF1 promoter in 78S and 78R cells. [file 12943_2022_1549_MOESM1_ESM.zip › Fig.S1.tif]

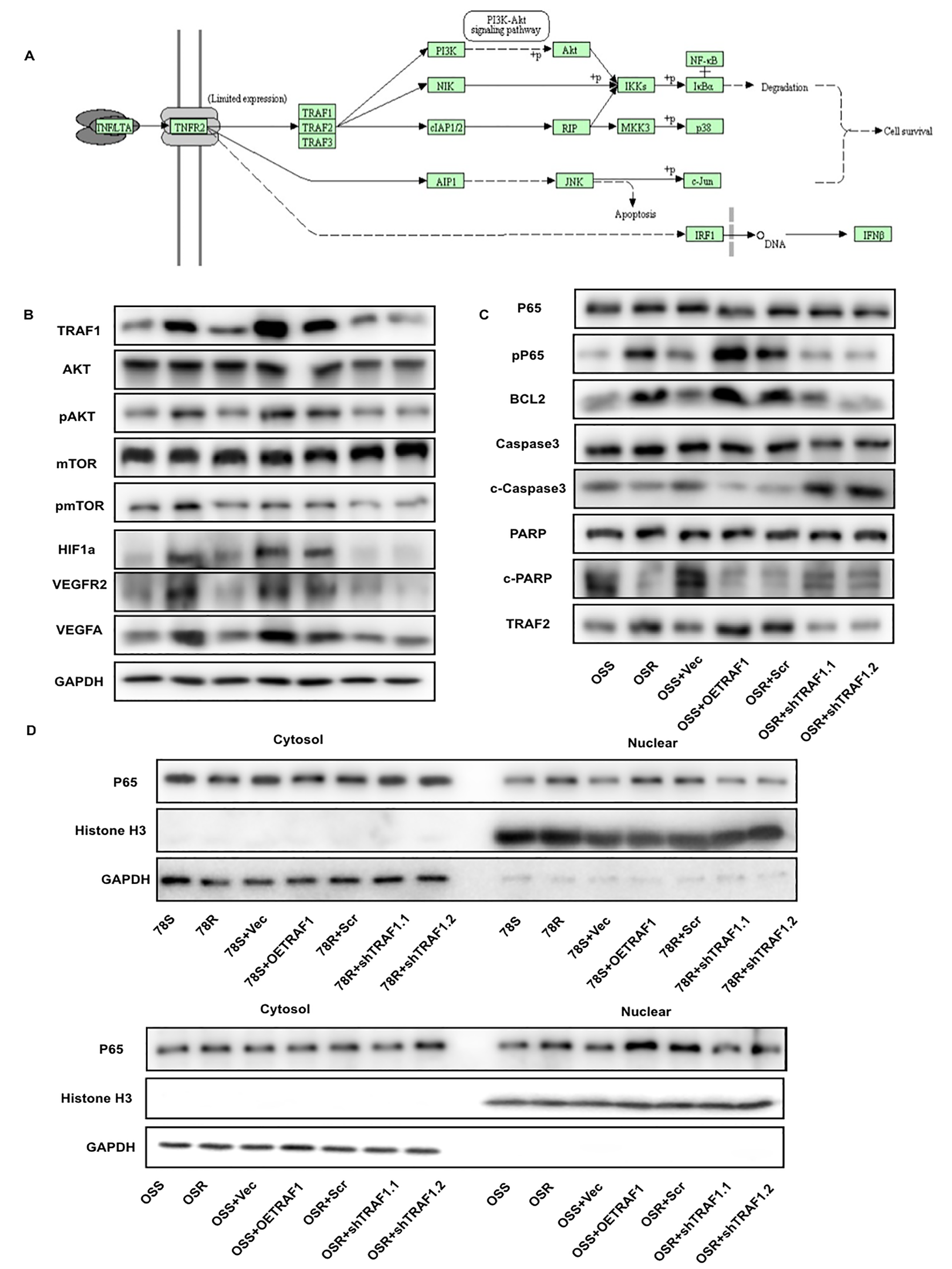

Supplement: Supplementary file 1 — Additional file 1: Table S2. Sequences of shRNA&siRNA against specific target in this study. Fig. S1 A Colony formation assay of sunitinib-resistant cell lines and control cell lines with DMSO in 12-well dish for 3 weeks (n = 3). Fig. S2 A TRAF1 pathways in KEGG. B Proteins involed in angiogenesis signaling were mediated by TRAF1 in OS-RC-2 cells. Fig. S3 A and B ChIP assays were used to assess the degree of H3K4me3 within the regions 1-3 of the TRAF1 promoter in 78S and 78R cells. [file 12943_2022_1549_MOESM1_ESM.zip › Fig.S2.tif]

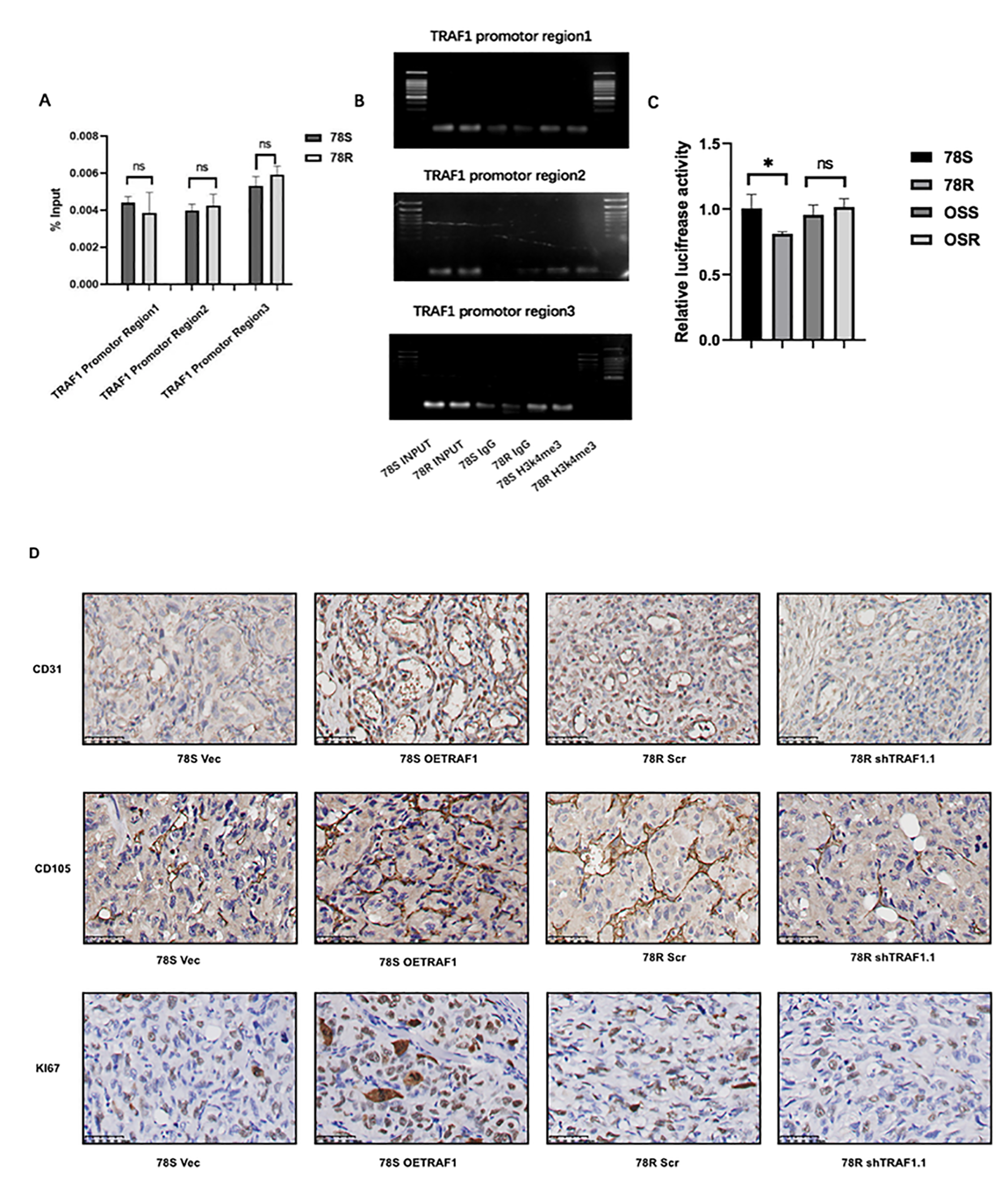

Supplement: Supplementary file 1 — Additional file 1: Table S2. Sequences of shRNA&siRNA against specific target in this study. Fig. S1 A Colony formation assay of sunitinib-resistant cell lines and control cell lines with DMSO in 12-well dish for 3 weeks (n = 3). Fig. S2 A TRAF1 pathways in KEGG. B Proteins involed in angiogenesis signaling were mediated by TRAF1 in OS-RC-2 cells. Fig. S3 A and B ChIP assays were used to assess the degree of H3K4me3 within the regions 1-3 of the TRAF1 promoter in 78S and 78R cells. [file 12943_2022_1549_MOESM1_ESM.zip › Fig.S3.tif]
