# Supplementary material for: N6-methyladenosine-modified TRAF1 promotes sunitinib resistance by regulating apoptosis and angiogenesis in a METTL14-dependent manner in renal cell carcinoma
Source: Mol Cancer. 2022 May 10;21:111. doi: 10.1186/s12943-022-01549-1 (PMC9087993; doi:10.1186/s12943-022-01549-1)
Supplement: Supplementary file 2 — Additional file 2. [file 12943_2022_1549_MOESM2_ESM.docx]

TRAF1

NM_005658-3utr(1870-2288nt)

GGCTGGCCATCTGGTTAGGATGGCAGGACGTGGGCTGGGCCCACAAAGGCAAAGGGTCCAGAAGGAGACAGGCAGAGCTGCTCCCCTCTGCACGGACCATGCGACACTGGGAGGCCAGTGAGCCACTCCGGCCCCGAATGTTGAGGTGGACTCTCACCAAATGAGAAGAAAATGGAACCAGGCTTGGAACCGTAGGACCCAAGCAGAGAAGCTCTCGGGCTAGGAAGATCTCTGCAGGGCCGCCAGGGAGACCTGGACACAGGCCTGCTCTCTTTTTCTCCAGGGTCAGAAACAGGACCGGGTGGAAGGGATGGGGTGCCAGTTTGAATGCAGTCTGTCCAGGCTCGTCATTGGAGGTGAACAAGCAAACCCAGACGGCTCCACTAGGACTTCAAATTGGGGGTTGGATTTGAAGACTT

NM_005658-3utr(1870-2288nt)-m6Amut

GGCTGGCCATCTGGTTAGGATGGCAGGACGTGGGCTGGGCCCACAAAGGCAAAGGGTCCAGAAGGAGACAGGCAGAGCTGCTCCCCTCTGCACGGCCCATGCGACACTGGGAGGCCAGTGAGCCACTCCGGCCCCGAATGTTGAGGTGGCCTCTCACCAAATGAGAAGAAAATGGAACCAGGCTTGGAACCGTAGGACCCAAGCAGAGAAGCTCTCGGGCTAGGAAGATCTCTGCAGGGCCGCCAGGGAGACCTGGACACAGGCCTGCTCTCTTTTTCTCCAGGGTCAGAAACAGGACCGGGTGGAAGGGATGGGGTGCCAGTTTGAATGCAGTCTGTCCAGGCTCGTCATTGGAGGTGAACAAGCAAACCCAGACGGCTCCACTAGGCCTTCAAATTGGGGGTTGGATTTGAAGACTT
