# Supplementary material for: N6-methyladenosine-modified TRAF1 promotes sunitinib resistance by regulating apoptosis and angiogenesis in a METTL14-dependent manner in renal cell carcinoma
Source: Mol Cancer. 2022 May 10;21:111. doi: 10.1186/s12943-022-01549-1 (PMC9087993; doi:10.1186/s12943-022-01549-1)
Supplement: Supplementary file 3 — Additional file 3: Table1. TRAF1 intensity of each patients. [file 12943_2022_1549_MOESM3_ESM.docx]

| Patient ID(sensitive) | TRAF1 intensity | Patient ID(resistant) | TRAF1 intensity |
| --- | --- | --- | --- |
| 1 | High positive | 1 | High positive |
| 2 | Positive | 2 | High positive |
| 3 | Positive | 3 | High positive |
| 4 | Low positive | 4 | Low positive |
| 5 | Low positive | 5 | Low positive |
| 6 | Low positive | 6 | Low positive |
| 7 | Low positive | 7 | Low positive |
| 8 | Low positive | 8 | Low positive |
| 9 | Negative | 9 | Positive |
| 10 | Negative | 10 | Positive |
| 11 | Negative | 11 | Positive |
| 12 | Negative | 12 | Positive |
| 13 | Negative | 13 | Positive |
| 14 | Negative | 14 | Positive |
| 15 | Negative | 15 | Positive |

Supplementary table1 TRAF1 intensity of each patients
